# Disparities in travel times between car and transit: Spatiotemporal patterns in cities

**DOI:** 10.1038/s41598-020-61077-0

**Published:** 2020-03-04

**Authors:** Yuan Liao, Jorge Gil, Rafael H. M. Pereira, Sonia Yeh, Vilhelm Verendel

**Affiliations:** 1 Department of Space, Earth and Environment, Division of Physical Resource Theory, Chalmers University of Technology, Gothenburg, 41296 Sweden; 2 Department of Architecture and Civil Engineering, Division of Urban Design and Planning, Chalmers University of Technology, Gothenburg, 41296 Sweden; 3 Institute for Applied Economic Research (Ipea) - Brazil, Department of Urban, Regional and Environmental studies and policies (DIRUR), Brasilia, 70076-900 Brazil; 4 Department of Computer Science and Engineering, Chalmers University of Technology, Gothenburg, 41296 Sweden

**Keywords:** Civil engineering, Information technology, Scientific data, Statistics

## Abstract

Cities worldwide are pursuing policies to reduce car use and prioritise public transit (PT) as a means to tackle congestion, air pollution, and greenhouse gas emissions. The increase of PT ridership is constrained by many aspects; among them, travel time and the built environment are considered the most critical factors in the choice of travel mode. We propose a data fusion framework including real-time traffic data, transit data, and travel demand estimated using Twitter data to compare the travel time by car and PT in four cities (São Paulo, Brazil; Stockholm, Sweden; Sydney, Australia; and Amsterdam, the Netherlands) at high spatial and temporal resolutions. We use real-world data to make realistic estimates of travel time by car and by PT and compare their performance by time of day and by travel distance across cities. Our results suggest that using PT takes on average 1.4–2.6 times longer than driving a car. The share of area where travel time favours PT over car use is very small: 0.62% (0.65%), 0.44% (0.48%), 1.10% (1.22%) and 1.16% (1.19%) for the daily average (and during peak hours) for São Paulo, Sydney, Stockholm, and Amsterdam, respectively. The travel time disparity, as quantified by the travel time ratio $R$ (PT travel time divided by the car travel time), varies widely during an average weekday, by location and time of day. A systematic comparison between these two modes shows that the average travel time disparity is surprisingly similar across cities: $R < 1$ for travel distances less than 3 km, then increases rapidly but quickly stabilises at around 2. This study contributes to providing a more realistic performance evaluation that helps future studies further explore what city characteristics as well as urban and transport policies make public transport more attractive, and to create a more sustainable future for cities.

## Introduction

Increased car use has many negative environmental impacts, including traffic congestion, land-use issues such as parking, increased air pollution, and greenhouse gas (GHG) emissions. Public transit (PT) can provide a low-cost, energy-efficient, less polluting, and socially equitable travel alternative^1,2^. Policymakers in many places worldwide recognise the importance of promoting a mode shift from car to PT in cities as a way to address negative environmental impacts, increase equity^3^, and combat climate change^4^. For instance, Berlin, Germany, is committing 28 billion euros from 2019 onward to improve PT^5^.

However, increasing PT ridership and reducing private car use have proven difficult. Despite significant investments, PT ridership has fallen steadily in the United States and elsewhere^6,7^. Growth in PT ridership is constrained by many factors, such as fixed schedules and routes, low population density, and travellers’ attitudes^8^. Among these constraints, travel time and the built environment are considered the most critical factors in the choice of transport mode^6,9,10^. A primary driver of PT ridership growth is the reduction of users’ perceived marginal cost, including travel time^11–13^. Travel time is also a key performance indicator for the quantification of PT service quality^14^. In a review paper, Redman *et al*. summarised studies of PT improvement strategies targeting different quality attributes^15^ and found that most studies focused on speed as a critical factor for increasing PT ridership. In a New York study, a 15-minute shorter commuting time corresponded to about 25% higher ridership for a rail service^15^. According to a post-trip questionnaire survey of car users, shorter travel time is one of the key factors that would make PT more attractive^16^.

While there is strong evidence that car-based travel is often faster than public transit, the spatial and temporal patterns of this time discrepancy are crucial to better inform urban planning and policy efforts to encourage travel mode shifts. Recent studies show how transit travel times can vary greatly by route and time of day^17,18^, while a growing body of research using GPS and mobile phone data to record travel speed shows that overall transport performance varies significantly by time of day due to congestion in cities^19–21^. Traditional static views on travel time calculation have largely relied on simplistic assumptions, using constant vehicle speeds^22^ and overlooking how travel speeds vary over the course of the day^23^.

The growing body of literature in understanding the spatiotemporal disparities in travel times for cars and PT^24,25^ starts using detailed spatial data and time-varying transport data sets, which provides opportunities for a more realistic assessment of modal disparity on travel time in this study. Rapidly emerging data sources and geographical information systems (GIS) have significantly increased the availability and the amount of data sensed in urban transport systems^26,27^, such as traffic speed data, taxi GPS data^27^, and PT smart card data^28^. The availability of real-time traffic speed data enables more advanced traveller information systems for route choice and better-informed traffic planning^29^. New data sources, such as HERE Traffic^30^ with extensive coverage of cities in 83 countries to date^29^, can collect and provide information about real-time road speed, incidents, and accidents. The amount of available data and the level of spatial and temporal resolution allow more realistic estimates of travel time^21^. The rise of open data also supports more realistic travel time calculations. Open data standards such as General Transit Feed Specification (GTFS)^31^ and crowdsourcing initiatives such as OpenStreetMap (OSM)^32^ provide data that can be used to estimate PT travel times with the most up-to-date schedules^33^.

In accessibility studies, travel times by car and PT are typically estimated for hypothetical destinations or limited to locations with certain functions such as workplaces or hospitals^34,35^. With accessibility analysis, the actual travel demand between places is not the primary focus but rather the “potentials for accessibility” to travel from/to a fixed point and/or the locations of interest in a region. However, the population flows between places are critical for evaluating transport performance of the actual demand, especially given how both transport demand and transport services vary by place and time of day.

Despite a large and growing literature on the travel time disparity between car and PT using real-world measurements, it remains to be explored how such disparity varies when considering the real travel demand. A full and realistic understanding of the disparities in travel times between these two modes could help identify opportunities of where and when public transit is competitive (time-wise) with automobiles and shed light on the relative transportation disadvantage of members of the community who must depend on public transit. Large-scale, representative dynamic travel demand data are critically needed for a more realistic assessment of this time disparity.

When compared with other conventional/new data sources e.g. travel surveys^36,37^, traffic data^38^, and origin-destination (OD) matrix, geotagged social media data (e.g. tweets with GPS coordinates specifying location) have been found to be a useful proxy for travel demand^37,39^. Unlike OD matrices that usually capture daily averages, for example, Twitter data, specifically the density of geotagged tweets, reasonably capture an accurate representation of where and when people are engaging in various activities with high spatiotemporal resolution, therefore making it a good and low-cost source for obtaining dynamic travel demand in cities.

This study leverages multiple large-scale data sources to capture, at a fine resolution, the spatiotemporal patterns of how car and PT travel times vary in four different cities: São Paulo, Brazil; Stockholm, Sweden; Sydney, Australia; and Amsterdam, the Netherlands. This study calculates the detailed spatiotemporal variations of travel times for an average weekday to improve the level of resolution at which we can understand the disparity in travel times between PT and car. We combine multiple data sources: HERE Traffic data over one year to derive empirical road speed, Twitter data accumulated from the past nine years, up-to-date GTFS transit data, and road networks from OpenStreetMap. Each city is divided into a hexagonal grid system, and travel times are estimated at different times of the day for any cell within the system (for more details, see Methods), calculating the door-to-door travel times by car and by PT to any highly visited cell (destination), identified as such based on geotagged tweet volumes. Within a selected time interval (e.g., 8:10 am to 8:25 am), the average travel time of a given origin cell is defined as the mean value of the travel times from that origin to multiple destinations whose volumes of geotagged tweets are used as weights. To quantify the modal disparity of travel time, we use the travel time ratio ($R$), defined as the travel time by PT divided by the travel time by car for a given origin-destination pair at a certain departure time. Finally, we visualise and analyse the results to demonstrate how car and PT travel times vary spatiotemporally across all the cities studied. Lastly, we present a systematic cross-regional comparison of the travel time disparity between car and PT in the four cities studied.

## Results

### Spatiotemporal variations of travel times

**Figure 1:**
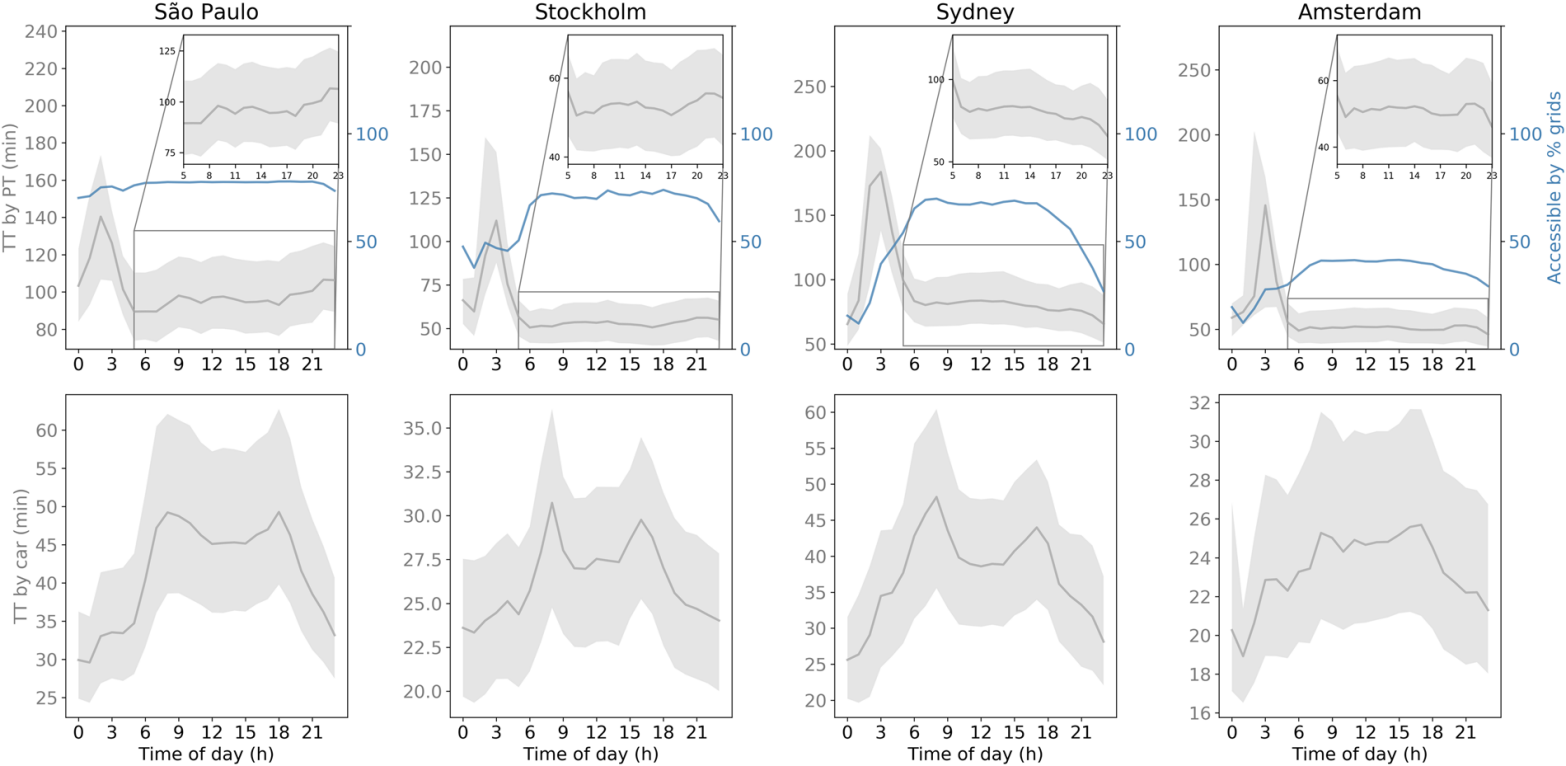
Temporal variations of travel time by public transit (upper row) and by car (bottom row) over the course of an average weekday. The travel time is the citywide average across departure locations, weighted by population density, of the average travel times from those locations. The shaded area indicates the range from the 25th to 75th percentile. Also shown is the percentage of grid cells accessible by PT by time of day. The inset figures are zoomed into the time period of from 05 hours to 23 hours to better show the variation of the travel time by PT.

**Figure 2:**
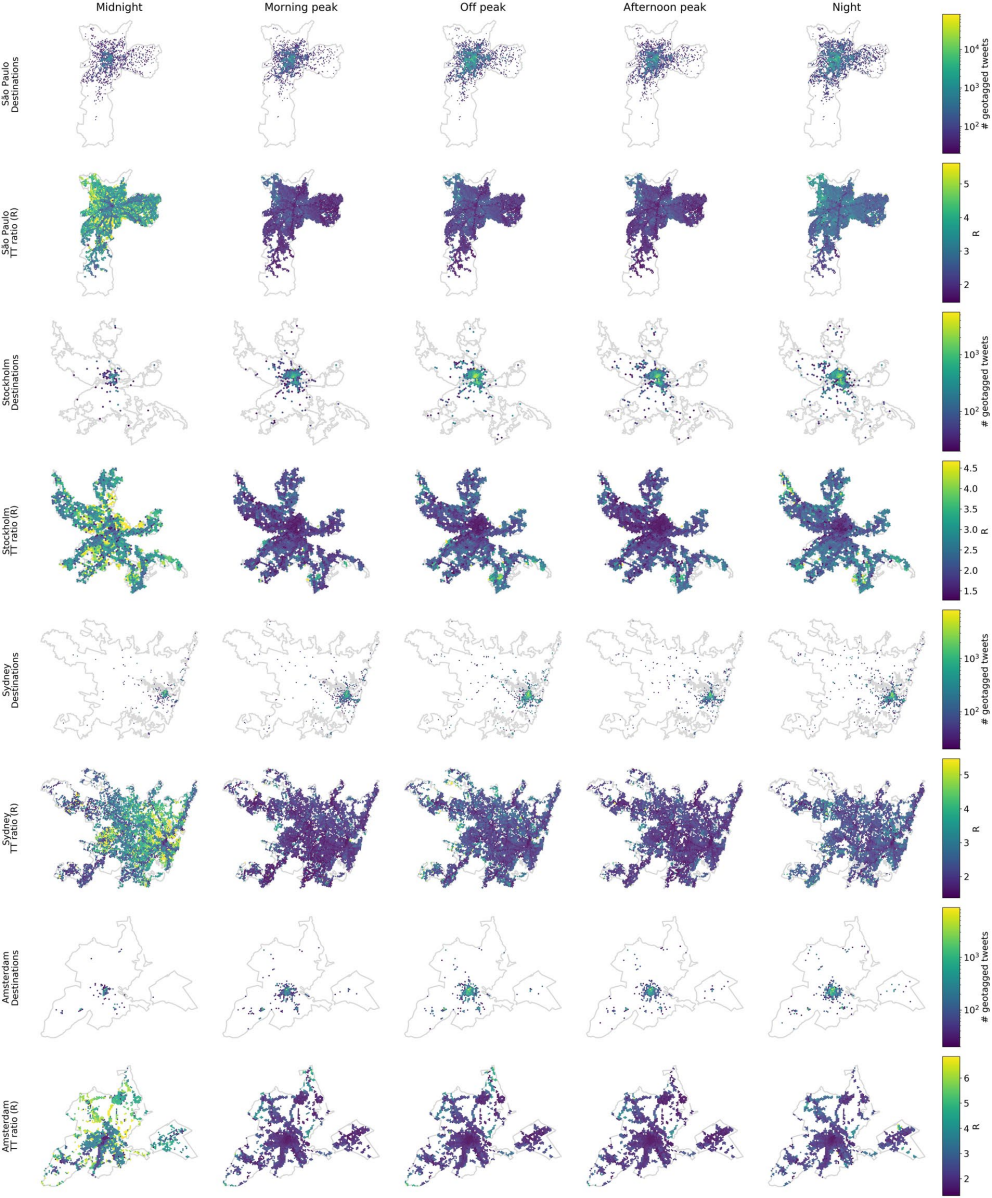
Spatiotemporal variation of travel time ratio ($R$) over the course of an average weekday. The travel time ratio $R$ is the ratio of the average PT travel time to the average car travel time. For each city, the upper row shows the visited locations weighted by their number of geotagged tweets, and the bottom row shows the weighted average travel time to those locations within each time interval. Midnight = 0:00–7:00, Morning peak = 7:00–10:00, Off peak = 10:00–16:00, Afternoon peak = 16:00–19:00, Night = 19:00–0:00. Maps presented with different spatial scales for the sake of clarity.

The temporal variation of the citywide average travel times (see Methods) to frequently visited locations is illustrated in Fig. 1. The two transport modes display rather different temporal patterns. For car use, Sydney and Stockholm have two distinct peaks during morning and afternoon rush hours, while São Paulo and Amsterdam show elevated travel time during daytime, with less pronounced rush-hour peaks. For PT, the travel time is generally longer from midnight to dawn when services are suspended or infrequent. In São Paulo, close to 80% of the grid is accessible by PT. Stockholm, Sydney, and Amsterdam have less coverage, in succession. Sydney has the most time-variable coverage, measured by the change in the share of grid cells that can access the destinations over the course of a typical weekday. Figure 2 shows the spatiotemporal variation in the travel time ratio ($R$) for the origin locations in each city. During peak hours, $R$ varies less with location than at other times of day. In Amsterdam, the maximum share of grid cells that can access the destinations is around 40% which is much lower than the other cities (Fig. 1). This is a result of the polycentric urban structure with PT provision concentrated along corridors in Amsterdam (Fig. 2).

**Figure 3:**
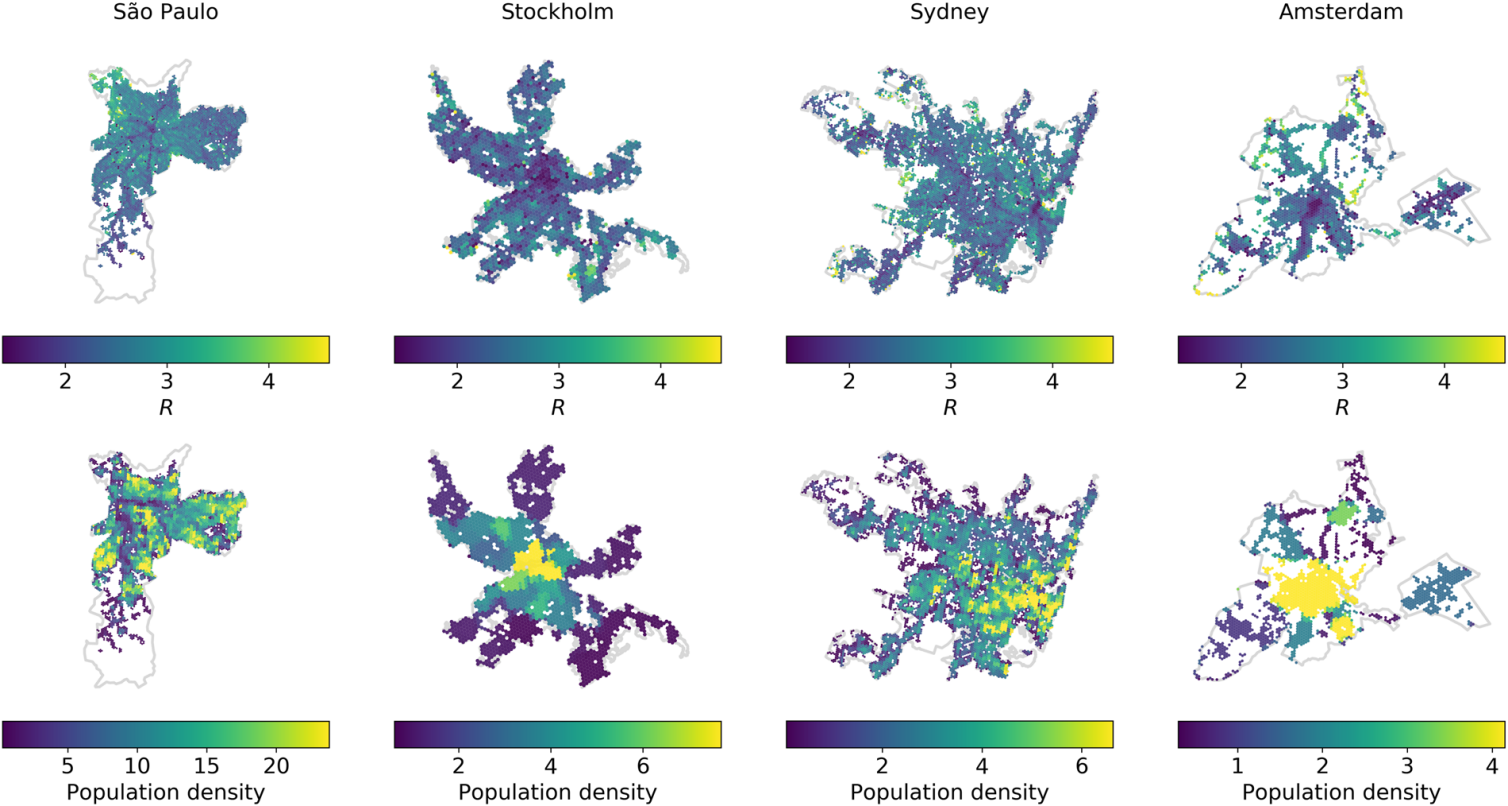
Spatial variation of travel time ratio ($R$) to frequently visited locations (top row) and in contrast with the population density distribution (bottom row). The value of $R$ for each cell as the origin is the average value based on the 5–95% quantiles of travel times by PT and car in the time period between 05:00 and 23:00 weighted by the frequency of geotagged tweets in the destinations (same below). The warmer the colour, the greater the advantage of car use over PT. The unit of population density is 1000 persons per sq. km.

The travel time ratio $R$ in Fig. 3 shows the spatial distribution of daily average travel time (see Methods) by PT compared with car use in contrast with the population density distribution. For each grid cell, taking PT takes around twice as long as using the car for all the study areas. In all four study areas, the spatial distribution of $R$ reflects the spatial organisation of cities so that the advantage of car travel over PT tends to be smaller near city centres, in regions with higher population and activity densities, and closer to PT infrastructure.

### Cross-regional comparison of the modal disparity of travel time

**Figure 4:**
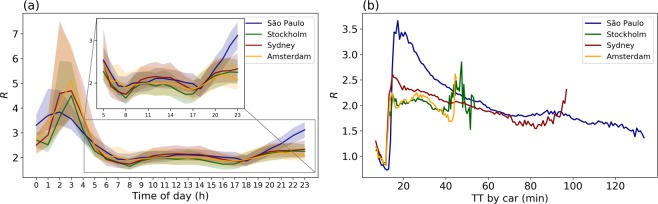
(**a**) Temporal variation of citywide average travel time ratio ($R$). The shaded area indicates the two mid quartiles. The insert zooms in on the period from 05 hours to 23 hours, to better show the temporal variation of $R$. (**b**) Travel time ratio ($R$) as a function of travel time by car.

The temporal variation of $R$ shown in Fig. 4(a) suggests that the average travel time ratio is around 2 throughout most of the day, and the highest disparity between the two modes occurs between midnight and before dawn, when PT service is typically reduced or not running at all. The share of area that favours PT over car use is very small: 0.62%, 0.44%, 1.10% and 1.16% (daily average) or 0.65%, 0.48%, 1.22% and 1.19% (during peak hours) for São Paulo, Sydney, Stockholm, and Amsterdam, respectively. Figure 4(b) shows how the travel time ratio changes as travel time by car increases. It turns out that PT outperforms cars on shorter trips, for trips with car travel times larger 18 min the disparity increases in favour of the car, and for longer trips the disparity between the two modes slowly decreases.

**Figure 5:**
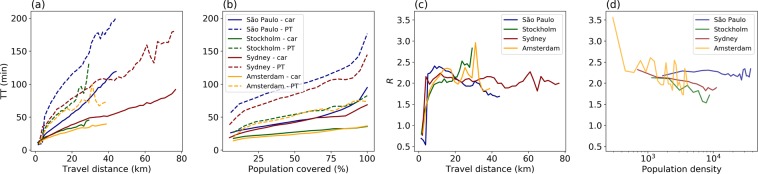
Travel time and travel time ratio vs. travel distance and population density. Travel time as a function of (**a**) travel distance and (**b**) the percentage of population reached. The relationship between travel time ratio ($R$) and (**c**) travel distance and (**d**) population density. The unit of population density is 1 person per sq. km.

Figure 5(a) shows the travel time by car and by PT as a function of travel distance (by car for the sake of comparison). Given the same travel distance, these cities have different travel times by car. São Paulo has the longest travel time by car for the same distance due to the high congestion levels^21^. Within the range of 0–40 km, the three other cities show similar levels of travel time by car, with Amsterdam having the lowest. Despite similar sizes, São Paulo and Sydney display different patterns of travel time by car with increasing travel distance: travel time in São Paulo increases with distance much more rapidly than in Sydney, presumably due to the high congestion levels. São Paulo also has the highest travel time with PT, whereas the three other cities have similar travel times by PT for the same distance. In general, travel time by PT increases quickly for short-distance trips (0–20 km) but increases at a lower rate for medium-distance trips (20–40 km). For long-distance trips (>30 km in São Paulo and >50 km in Sydney), the travel time once again becomes sensitive to the increased distance.

Figure 5(b)shows that the population can reach the frequently visited locations in the study areas by car faster than by PT, with travel time rising quickly and with a long tail in São Paulo and Sydney (i.e. the 10% of the population who live farthest away from the destinations have an average travel time that is roughly 3 times that of the closest 10%). In Fig. 5(c), $R$ can be less than 1 (PT faster than car use) for distances <3 km, but PT quickly loses the advantage as distances increase. Except for Stockholm, the cities show similar patterns when travel distances continue to increase: The disparity between PT and car travel time continues to increase until it reaches a maximum value at around 15 km, and then it starts to drop. This can be explained by the slower increase of travel time by PT than by car. In addition, as shown in Fig. 5(d), population density and $R$ are also correlated: The greater the population density, the lesser the disparity between PT and car travel times.

At the city-level, Table 1 summarises the performance of PT and car use in terms of travel time ratio and the aggregate travel speed. The mean value of the (unweighted) travel time ratio for the study areas is 2.2 (São Paulo), 2.0 (Stockholm), 2.2 (Sydney), and 2.2 (Amsterdam), where the smallest difference between the modes is observed in Stockholm. The travel time ratio $R$ weighted by population density is 1.4, 2.6, 2.3, and 2.1 for São Paulo, Stockholm, Sydney, and Amsterdam, respectively, suggesting PT services in São Paulo and Amsterdam are more closely matched with where people live versus the PT services in Stockholm and Sydney, which are focused more on spatial coverage. Although Stockholm has the smallest disparity between PT and car, when the average travel time ratio is weighted by the population density, Stockholm becomes the city with the largest disparity. However, São Paulo shows the opposite trend after incorporating population density; it becomes the city with the smallest disparity between PT and car. For PT, the differences of (population weighted) speed are small. For São Paulo, the low driving speed suggests heavy traffic congestion, explaining why the disparity in time between PT and car is smallest there.

**Table 1:** Travel time ratio at the city level.

City	Travel time ratio	Travel time ratio${}_{{\boldsymbol{pop}}}$^a^	Speed (${\boldsymbol{k}}{\boldsymbol{m}}{\boldsymbol{/}}{\boldsymbol{h}}$)		Speed${}_{{\boldsymbol{pop}}}$ (${\boldsymbol{k}}{\boldsymbol{m}}{\boldsymbol{/}}{\boldsymbol{h}}$)^b^	
			Car	PT	Car	PT
São Paulo	2.2	1.4	19.4	9.2	19.9	14.3
Stockholm	2.0	2.6	25.7	12.9	37.6	14.9
Sydney	2.2	2.3	33.8	16.6	30.9	13.9
Amsterdam	2.2	2.1	31.5	15.0	27.6	13.7

^a-b^Average value weighted by population density in each grid cell. The travel time ratio at the city level is calculated based on the average value across all grid cells at all times of the day weighted by the frequency of geotagged tweets of the destinations (see Methods).

## Discussion

Travel time itself is the core indicator in a wide variety of accessibility measurements in the literature which assesses how easily people can reach various destinations for different activities given a travel time budget by a certain mode^17,40^. However, varying travel demand can substantially alter travel time in urban settings when compared with theoretical estimates^41,42^. In this study, we provide a different perspective to assess the transport systems, focusing on the performance given the actual demand. With time-varying travel demand from Twitter data, our methodology connects transport service demand and operations, allowing for more realistic comparisons between the two modes (car and PT) in cities. The methodology reveals where the biggest gaps are and informs planners and policymakers of the areas where improvements would have the largest impact given today’s demand.

We propose a data fusion framework that is repeatable and can scale up, where we incorporate emerging data sources, including Twitter, HERE Traffic, OSM, and GTFS, to model travel time by car and by PT. HERE Traffic provides high-frequency data of the actual driving speed in the cities studied with extensive coverage. We use GTFS data plus a trip planner as an “advanced” method^42^ because GTFS data already takes into account congestion-related delays in route planning. In addition to real-time road speed records and GTFS data, that have been used in previous studies, we introduce social media data, in this case from Twitter, to represent the locations of actual demand by time of day for an average weekday, given the validity of using Twitter to represent the actual travel demand^36,37^. The usefulness of the framework is demonstrated by revealing the modal disparity of travel time at a high spatial and temporal granularity and how these patterns vary across different cities. The temporal and spatial variations in travel time for car and PT are described in detail for the four study areas (Fig. 1 and in Fig. 2). They show how the travel time for each mode changes by time of day for an average weekday and how travel time varies spatially in different cities. Future studies can zoom in and overlay infrastructure information to gain more detailed insights at the local level. This allows for urban planning policies to be better informed, especially in encouraging a mode shift from car to PT. While trips by PT take on average around twice as long as by car, this difference varies widely with location (Fig. 2) and time of day (Fig. 5). The travel time difference is consistent with previous studies, but this observation is now supported by better and more detailed spatiotemporal data and real travel demand.

We present how the spatiotemporal variation of travel time differs between cities, and we further explore the impact of travel distance and population density on travel times across cities (Fig. 5(a,b)). Travel times increase in different ways for cars and PT when the travel distance increases (Fig. 5a). For both cars and PT, São Paulo shows significantly longer travel times compared with the other cities. To cover the same percentage of the total population, taking PT takes significantly longer time than driving a car (around twice, see Fig. 5b). Moreover, for PT in the two big cities, São Paulo and Sydney, the final 10% of the population who live far away from the destinations have an average travel time that is roughly 3 times that of the first 10% (Fig. 5b).

In relation to the modal disparity in travel times and its relationship with travel distance and population density (Fig. 5c,d), the results are consistent with the commonsense expectation that short travel distance, city centres, and the proximity to PT lines are situations in which PT can outperform car use. Similar patterns have been found in previous studies, e.g. of Greater Helsinki^42^. However, it is important to recognise that only a very small number of grid cells where PT outperforms cars in terms of travel time. PT can outperform car use ($$R$$$$ < =$$ 1) for short distances (<3 km), mainly during rush hour in Stockholm and Amsterdam, when the share of grid cells that favour PT can reach 0.8% in Amsterdam and 0.6% in Stockholm. This number is even smaller in Sydney and São Paulo.

We summarise the city average of the modal disparity across four cities studied (Table 2). At the city level (with grid cells weighted by population density), the lowest travel time ratio is observed in São Paulo, followed by Amsterdam, Sydney, and Stockholm in ascending order. The heavy traffic in São Paulo explains the observed lowest travel time ratio. However, the heavy traffic also affects travel times for bus in reality. It has been shown that in São Paulo there are large inconsistencies between the scheduled PT and the actual location of their buses^43^, therefore, bus GPS data have been used to account for congestion. In this study, however, we did not include the effect of congestion on PT. Consequently, we may overestimate the advantage of PT over car use for São Paulo. For urban planners and policy-makers, such inconsistencies can be identified and solved with real-time schedule updates.

**Table 2:** Study cities’ main characteristics and mode share (D = Driving, PT = Public transit, W = Walking, B = Biking).

City	Scope	Area (km$${}^{2}$$)	Population	GDP ($/capita)	Mode share (%)^a^			
					D	PT	W	B
São Paulo	Municipality	1,521	11,967,825	9,812	28	31	31	1
Stockholm	Urban area^b^	414	1,372,565	53,253	32	47	14	7
Sydney	UCL^c^	2,036	4,321,535	49,755	59	25	4	3
Amsterdam	Urban Area^d^	885	1,520,127	45,638	20	17	29	32

^a^Sources: São Paulo, Sydney, and Amsterdam in 2019 (Deloitte City Mobility^67^), Stockholm in 2011 (National Travel Survey). ^b^Statistics Sweden - Open geodata for urban areas^68^. ^c^Urban Centres and Localities defined by Australian Bureau of Statistics - Significant Urban Areas, Urban Centres and Localities (UCL), Section of State, July 2011^69^. ^d^“Stadsgewesten” defined by Statistics Netherlands based on “Division of the Netherlands into 22 metropolitan agglomerations and urban areas” (2015)^70^.

Travel time has been found to be the strongest predictor of mode choice^44^, especially when PT is the alternative travel mode for car drivers^45^. In our study, the area in the studied cities where PT can outperform car use is very small, despite there also being substantial areas surrounding PT lines where the disparity of travel time by car and PT is smaller than in the rest of the city. A survey of car drivers conducted in Amsterdam showed that when the perceived travel time ratio dropped below 1.6, participants were willing to regularly make the trip by PT^45^. The average travel time of trips by car recorded in that survey was 35 min. However, our study shows that the travel time ratio is at best 2, for trips with car travel time of around 35 min (see Fig. 4b). When asked “Could you also have made this trip by public transport?” in relation to a 35-minutes trip, prompts car drivers to say “yes, but rarely do” when the ratio is 2. To get more car drivers to take PT, the travel time by PT needs to decrease further. However, the time elasticity of PT is greater than for cars, which means that to increase PT ridership, the marginal decrease of travel time is lower compared to for cars^46^. Travel time alone does not tell the whole story when encouraging a mode shift from car to PT, and PT being as fast as a car would not mean that everyone would prefer to take it^47^. Many other factors contribute to mode choice, e.g. comfort and price.

## Conclusion

To assess the difference in travel time between car use and PT, we propose a computational framework incorporating new and large data sets, especially social media data to represent the locations of actual demand by time of day for an average weekday. We implement this framework in four cities and make a systematic comparison across them. The framework demonstrates its usefulness by revealing the travel time disparity between PT and cars at a high spatial and temporal granularity enabling detailed and local-level explorations. On average, PT travel time is around twice as high as by car, confirming previous studies, but with more detailed real-world travel demand data. The disparity tends to be much smaller near city centres and in the surroundings of PT lines. PT can outperform car use on average for short-distance travel (<3 km) and during peak rush hour in Stockholm and Amsterdam. A systematic comparison between these two modes shows that the travel time disparity is surprisingly similar across cities: $$R\,$$$$ < $$ 1 for travel distances less than 3 km, then increases rapidly but quickly stabilises at around 2.

To better compare car with PT in future studies, one can include more features from the geographical context, consider the effect of congestion on PT, and discuss the environmental performance, especially the GHG performance, of each mode by time of day, location and travel distance.

## Methods

**Figure 6:**
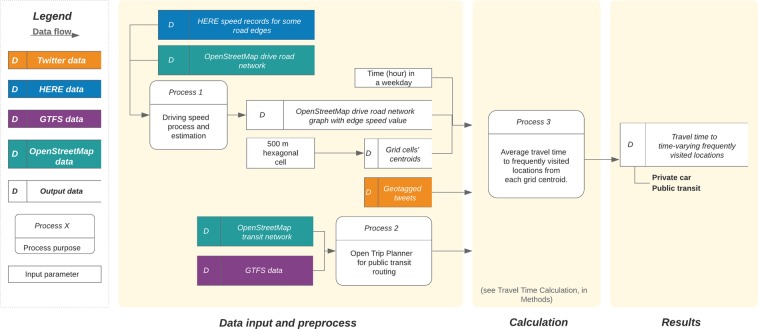
Flowchart of the analytic framework used for calculating the real-world travel time by car and PT.

The framework of data analysis is shown in Fig. 6. We combine four big data sources in this study: HERE Traffic data, Twitter geotagged tweets, OSM road network, and GTFS data. Each city’s study area is divided into hexagonal cells with a short diagonal of 500 m. All grid cells are represented by the latitude/longitude coordinates of their centroids. Geotagged tweets are used to derive the frequency of visited locations in each city. Some studies find good agreement, in general, between geotagged tweets and either traffic data^38^ or travel-demand data^36^. In addition, geotagged tweets offer realistic activity (i.e. demand) locations^37^ to avoid having to estimate the travel time for all potential pairs of origin-destination in the city^42^. We classify locations visited frequently, i.e. those with more than 20 geotagged tweets over each hourly interval, as “destinations” and calculate the travel time by car and PT from everywhere in the city to these destinations, see Travel Time Calculation.

### Data and preprocessing

#### HERE traffic data: driving speed

Data on driving speed were collected approximately every 5 minutes from January 1, 2018, to December 31, 2018. In this study, we focus on an average weekday. Therefore, we remove the data collected in January, February, July, August, and December to eliminate the impact of holiday seasons. Because of the variations in the timing of the sampling and varying latency in network delay, these samples are grouped into 15-minute time windows (96 windows per day), in which the measured traffic speeds in each bin are averaged. Roads are geographically represented by road segments as a sequence of edges. We calculate the hourly average speed for each segment over weekdays. More details of the preprocessing method can be found in^21^.

#### GTFS Data: Public Transit Timetables

A GTFS static dataset^48^ is a collection of text files consisting of all the information required to reproduce a transit agency’s schedule, including the locations of stops and timing of all routes and vehicle trips. GTFS data were collected from various sources that are publicly available. São Paulo’s GTFS data were obtained from OpenMobilityData^49^ provided by SPTrans, Stockholm’s from OpenMobilityData provided by Stockholm SL, Sydney’s from the Open Data portal provided by the Transportation Department of New South Wales^50^, and Amsterdam’s from the Open Data portal of 9292^51^.

#### OSM data: road network for driving

To calculate travel time by car, the road network is downloaded using osmnx^52^ by specifying the road network type as “drive” and the area as per the study areas. The road network is then converted to a directed graph^52^ and further converted into an igraph object^53^. The road network is also necessary for the selection of walking to and from PT stops. To calculate travel time by PT, the OSM data files are downloaded at the country level from Geofabrik^54^, and the cities’ OSM data are further extracted using their corresponding poly files^55^.

#### Twitter data: time-varying travel demand

To identify non-commercial geotag users who geotweet frequently, we use the Gnip database purchased from Twitter (20 December 2015–20 June 2016) within the geographical bounding box of cities or countries of our study^56^; Gnip is a Twitter subsidiary which sells historical tweets in bulk, and provides access to the Firehose API^57^. Top geotag users are selected if having at least 50 geotagged tweets per year. We extract these top geotag users’ historical tweets occurring within the study areas using the User Timeline API^58^. The obtained geotagged tweets are further processed to represent the frequently visited areas in the cities. A more detailed description of the preprocessing methods of geotagged tweets and their validity in representing population-level demand can be found in our previous study^37^.

For each hourly interval during weekdays, the frequently visited locations are represented by the centroids of each grid cell for which there are at least 20 geotagged tweets captured in that hour. The total numbers of geotagged tweets and users for each city are summarised in Data Description, below.

#### Data description

The summary statistics of the four cities are shown in Table 2. For São Paulo, the study area is the boundary of the municipality due to the lack of data for the metropolitan area. For the other cities, the study area is the functional urban area.

**Figure 7:**
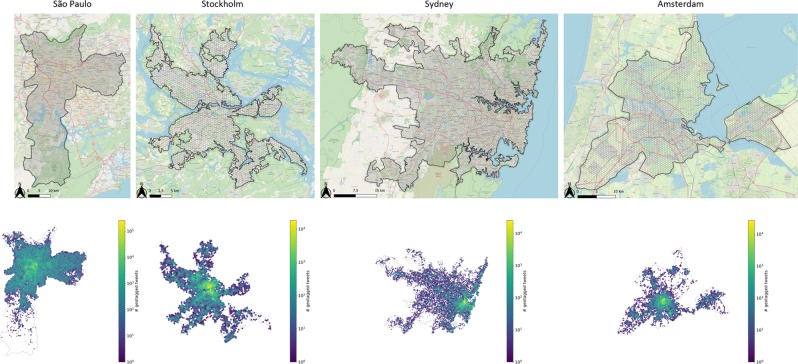
Studied cities: defined urban area (upper row) and spatial distribution of geotagged tweets (bottom row, all records are weekdays only).

Basic information on the data sets collected for the four cities is shown in Table 3. The study areas and the spatial distribution of geotagged tweets are shown in Fig. 7. In addition, we include population density data in the analysis^59^. Population density is used for visual comparison with the spatial distribution of travel time by different modes, and also used to weight the origin cells when aggregating the results across different grid cells for the travel time ratio ($R$) at the city level.

**Table 3:** Basic information of the data sets in this study.

City	Grid cells #	HERE^a^ (%)	GTFS (Date)	Twitter		
				User #	Geotag #	Time span^b^
São Paulo	7,423	55	2019-05-10	21,522	2,511,710	2010-09–2019-06
Stockholm	2,543	48	2019-05-15	5,344	310,609	2010-09–2019-03
Sydney	10,564	51	2018-05-14	11,707	608,699	2010-09–2019-05
Amsterdam	4,638	73	2018-05-28	9,424	284,281	2010-09–2019-04

^a^HERE indicator shows the share of real-time records. ^b^Time span shows the time period from the earliest timestamp to the latest timestamp of geotagged tweets in the collected user timelines.

### Travel time calculation

#### Origins, destinations, and travel time

We calculate the travel time of fastest routes from the geographical centre of all grid cells to the frequently visited destinations indicated by geotagged tweets in the city. The destination cells vary depending on the time of day, aggregated on an hourly basis. During a certain time interval $t$ (e.g., 8 am to 9 am), the average travel time of a given origin cell $i$, ${T}_{mode}(i,t)$, is defined as the mean value of the travel times from that origin to multiple destinations indexed as $j=1,2,...,n(t)$ whose frequency of geotagged tweets $f(j,t)$ are used as weights:

1 $${T}_{mode}\left(i,t\right)=\frac{{\sum }_{j=1}^{n(t)}\left[\,f\left(j,t\right)\cdot {T}_{mode}\left(i,j,t\right)\right]}{{\sum }_{j=1\,}^{n(t)}f\left(j,t\right)}$$

where ${T}_{mode}(i,j,t)$ indicates the fastest travel time by a certain mode from origin cell $i$ to destination cell $j$.

The average travel time over an average weekday is defined as:

2 $${T}_{mode}\left(i\right)=\frac{{\sum }_{t=0}^{24}{\sum }_{j=1}^{n(t)}\left[\,f\left(j,t\right)\cdot {T}_{mode}\left(i,j,t\right)\right]}{{\sum }_{t=0}^{24}{\sum }_{j=1}^{n(t)}\,f\left(j,t\right)}$$

At the city level, the average travel time is calculated as:

3 $${T}_{mode}=\frac{{\sum }_{t=0}^{23}{\sum }_{i=1}^{N(t)}{\sum }_{j=1}^{n(t)}\left[\,f\left(j,t\right)\cdot {T}_{mode}\left(i,j,t\right)\cdot Pop\left(i\right)\right]}{{\sum }_{t=0}^{23}{\sum }_{i=1}^{N(t)}{\sum }_{j=1}^{n(t)}\,f\left(j,t\right)\cdot Pop\left(i\right)}$$

where $N(t)$ represents the total number of hexagonal cells that can access the destinations at the given time frame and $Pop\left(i\right)$, when applied, indicates the population density of the origin cell $i$.

The detailed methods of calculating travel time by car ${T}_{car}\left(i,j,t\right)$ and by PT ${T}_{PT}\left(i,j,t\right)$ are shown in the next two sections.

#### Travel time by car

We calculate the travel time by car through real-time road speed records from HERE Traffic and speed limits of downloaded drive road networks from OSM. Not every road in OSM is covered by HERE Traffic data. For roads that are not, the average driving speed is estimated based on the average speed for the same road type (“highway” tag in OSM) derived from HERE Traffic data, if applicable. For those roads that do not have real-time records or road-type average, the speed is estimated based on the speed limit in OSM if applicable. The speed of 30 km/h is assigned to any remaining road edges, which account for less than 0.05% of the total road edges.

Once the road speed is processed, a directed graph is constructed from the “drive” road network data, with the edges assigned a travel time based on the average speed and the length of that road edge. We calculate the travel time of each road edge with the departure time ranging from 00:00, at an hourly frequency, until 23:00 (included) on weekdays. The origin and destination are the nearest road nodes to the respective cells’ centroids, and the impedance for routing is the travel time of the road edge. The travel time from a given origin node to a given destination node is represented by the travel time of the fastest route identified using Dijkstra’s algorithm^60^ plus a random value between 5 and 10 minutes as the estimated time for parking following the method adopted in previous studies^61^.

#### Travel time by public transit

Travel time by PT is calculated based on GTFS data and OSM data on a normal weekday. Due to varying data availability across the study cities, we select the first Wednesday in May after the updating date of GTFS as shown in Table 3. Routing between a given origin-destination pair is conducted using an open-source multi-modal routing engine, OpenTripPlanner (OTP)^62^, similarly used in previous studies^18,63,64^.

A trip by PT potentially consists of all available modes of public transportation (bus, tram, train, subway, etc.) and walking (speed = 1.4 m/s). For each pair of origin-destination, OTP finds the fastest door-to-door trip given a set departure time and the combination of transport modes available. The maximum walking distance is set to 800 m. To better balance the errors and the computation time, we use a hybrid sampling approach with 15-min resolution that helps us avoid the Modifiable Temporal Unit Problem (MTUP)^18,35^. The departure times are set to every 15 min from a randomly selected start time until an average weekday is sampled by 96 evenly spaced times. A grid cell is marked as “not reachable by PT” if the fastest route between two locations does not meet the constraint of maximum walking distance of 800 m or if the trip takes longer than 240 minutes.

#### Relative travel time by car use vs. PT

We propose a travel time ratio calculated for grid cell $i$ as the origin to the destination cell $j$ quantifying the disparity between these two modes as shown below:

4 $$R\left(i,j,t\right)={T}_{PT}\left(i,j,t\right)/{T}_{car}\left(i,j,t\right)$$

In this equation $R\left(i,j,t\right)$ is the ratio between travel time by public transit ${T}_{PT}\left(i,j,t\right)$ for an origin-destination pair ($i-j$) and the travel time by car ${T}_{car}\left(i,j,t\right)$ for the same origin-destination pair ($i-j$). The higher the $R$, the less desirable PT is compared to the car.

It is worth noting that we are comparing the empirically estimated travel time by car with the scheduled PT travel time. While GTFS data normally takes recurrent congestion into account, previous studies have shown that scheduled transit services can be affected by unplanned factors such as traffic accidents and weather conditions, causing some deviations from the schedule^65,66^. Nonetheless, it was not possible to find GPS data for all the public transport services analysed in this paper to compensate for deviations from the schedule. The use of GTFS data that already incorporates information on chronic congestion levels, combined with the adopted hybrid sampling approach of departure times, should minimise eventual measurement issues. If anything, this limitation would give an underestimate of the travel time gap between car and public transit.

When calculating the average values of $R$ across different times or grid cells, the same aggregation method is applied as demonstrated in Eqs. (1)–(3).
